# Detection of endosymbiotic, environmental, and potential bacterial pathogens in diverse mosquito taxa from Colombian tropical forests using RNAseq

**DOI:** 10.3389/fmicb.2025.1727830

**Published:** 2025-12-17

**Authors:** Cristian Robayo-Cuevas, Howard Junca, Sandra Uribe, Andrés Gómez-Palacio

**Affiliations:** 1 Laboratorio de Investigación en Genética Evolutiva – LIGE, Universidad Pedagógica y Tecnológica de Colombia, Tunja, Colombia; 2 RG Microbial Ecology, Metabolism, Genomics and Evolution, Division of Ecogenomics and Holobionts, Microbiomas Foundation, LT11A, Chia, Colombia; 3 Grupo de Investigación en Sistemática Molecular - GSM, Departamento de Biociencias, Facultad de Ciencias, Universidad Nacional de Colombia, Medellín, Colombia; 4 Grupo de Estudios en Genética y Biología Molecular – GEBIMOL, Universidad Pedagógica y Tecnológica de Colombia, Tunja, Colombia

**Keywords:** bacterial communities, metatranscriptome, Aedini, Culicini, Sabethini, Orthopodmyiini

## Abstract

**Introduction:**

Mosquitoes of the subfamily *Culicinae* transmit pathogens of major medical and veterinary importance, particularly in tropical regions where urbanization and ecological change promote arbovirus circulation. In Colombia, rural *Culicinae* species are diverse and harbor microbiomes that may influence vector competence, yet their bacterial communities remain poorly characterized.

**Methods:**

We characterized the bacterial microbiota of multiple *Culicinae* species and morphotypes collected from two rural localities in Antioquia, Colombia, using an integrated metagenomic approach. Ribosomal 16S rRNA sequences were extracted from total RNA-seq datasets to infer bacterial community composition and assess *α*- and *β*-diversity. Diversity metrics (Chao1 and Shannon indices), Discriminant Analysis of Principal Components (DAPC), and Bray–Curtis ordination were used to evaluate community structure. In parallel, *de novo* assembled contigs were taxonomically annotated against the NCBI NR bacterial database to obtain complementary taxonomic and functional insights.

**Results:**

Culex morphotypes exhibited the highest richness and evenness, whereas *Aedes* and *Trichoprosopon* showed lower diversity. Ordination and DAPC analyses revealed partial clustering by species and tribe. Both the 16S and assembly-based analyses showed complex bacterial assemblages dominated by *Wolbachia* (up to 60% of reads in several *Aedes* and Culex morphotypes), followed by environmental genera such as *Pseudomonas* and *Acinetobacter* (10–20%). Lower-abundance taxa of medical and veterinary importance—including *Salmonella*, *Borrelia*, and *Clostridium* (<5%)—were also detected. Bacterial community structure differed among mosquito species; *Aedes albopictus* was enriched in lactic acid bacteria, while *Culex* morphotypes exhibited broader environmental and endosymbiotic profiles.

**Discussion:**

This study provides the first comprehensive metagenomic description of bacterial communities associated with rural *Culicinae* mosquitoes in Colombia. The predominance of symbionts such as *Wolbachia* and *Spiroplasma*, coupled with distinct bacterial signatures among host species, highlights the ecological complexity of these microbiomes and their potential relevance for microbiome-based strategies in sustainable arboviral disease management.

## Introduction

Mosquitoes of the family Culicidae are hematophagous insects of global importance, acting as vectors of pathogens that affect both humans and wildlife (Becker et al., 2010). Genera such as *Aedes*, *Anopheles*, and *Culex* include species that are recognized as primary vectors of arboviruses including dengue virus (DENV), yellow fever virus (YFV), Zika virus (ZIKV), chikungunya virus (CHIKV), and West Nile virus (WNV), as well as parasites such as *Plasmodium* and filarial nematodes (Becker et al., 2010; Golding et al., 2015; Ethics and Vector-Borne Diseases: WHO Guidance, 2020). These pathogens impose a heavy public health burden, with dengue alone causing 50–100 million symptomatic infections annually and reaching record incidence in 2023 (Wilder-Smith et al., 2013; Cattarino et al., 2020; Moonen et al., 2023; Haider et al., 2025). Beyond pathogen transmission, mosquitoes host complex microbial communities that contribute to biological processes such as immune regulation, nutrient acquisition, reproduction, and vector competence.

Among these microbial partners, bacteria constitute a particularly dynamic component of the mosquito microbiome. Multiple studies have shown that bacterial taxa modulate susceptibility to arboviruses, either enhancing or restricting viral replication (Dong et al., 2009a; Coon et al., 2014a; Hegde et al., 2015; Guégan et al., 2018). For example, gut colonization by *Rosenbergiella* sp. blocks infection by both DENV and ZIKV in *Ae. aegypti* and *Ae. albopictus*, with stable transstadial transmission across life stages (Zhang et al., 2024). Similarly, symbionts such as *Asaia*, *Serratia*, and *Wolbachia* influence transmission dynamics through direct competition with pathogens or by modulation of host immunity (Caragata et al., 2016; Pan et al., 2018a). These findings highlight bacterial communities not only as a determinant of vector competence but also as a promising target for microbiome-based interventions.

In Colombia, more than 300 mosquito species across 28 genera have been documented, and nearly one quarter of the population lives in areas endemic for at least one mosquito-borne disease (Montoya-Lerma et al., 2011; Cano-Pérez et al., 2023). Environmental change—including urbanization, agriculture, deforestation, and climate warming—is expanding vector ranges and raising the risk of established and emerging pathogens. Regions such as Antioquia are particularly favorable for *Aedes aegypti* and *Culex* spp., while the coexistence of urban and sylvatic *Aedes* in Caribbean forest fragments underscores the potential for re-emergent arboviral threats (Ramasamy and Surendran, 2016; Cano-Pérez et al., 2023). Understanding the microbiome of sylvatic mosquito assemblages is therefore essential to unravel local vector biology and identify candidate symbionts for paratransgenic or microbiome-modulation strategies (Calle-Tobón et al., 2022; Gómez-Palacio et al., 2025).

Beyond medically relevant arboviruses, the mosquito virome also comprises insect-specific viruses (ISVs) and viruses associated with the microbiota, such as bacteriophages and fungi-related viruses. These viral communities play crucial roles in vector competence, environmental interactions, and evolutionary dynamics (Shi et al., 2020; Ramírez et al., 2024). ISVs, while restricted to invertebrates, share phylogenetic relationships with pathogenic arboviruses and are studied as models of restriction mechanisms and potential biocontrol agents (Vasilakis and Tesh, 2015; Zhang et al., 2024). Importantly, ISVs interact with other microbiome components, including bacteria and bacteriophages, generating multilayered networks that influence pathogen susceptibility. For instance, ISVs can modulate immune pathways that also regulate bacterial colonization, while phages shape bacterial communities in ways that affect viral replication. Together, virome and bacterial communities act as co-regulators of mosquito physiology and vector competence. Recognizing these cross-domain interactions is central to microbiome modulation, where viruses and bacteria may operate as synergistic levers for sustainable mosquito-borne disease control.

Our previous work in Colombia’s Northern Inter-Andean Valleys revealed a core set of ISVs and tribe-specific viral signatures in non-urban Culicinae, emphasizing the ecological distinctiveness of sylvatic mosquito viromes (Gómez-Palacio et al., 2025). Yet, the bacterial fraction of the microbiome remains comparatively understudied in these same populations. Given that bacteria can directly influence pathogen replication, immune activation, and host fitness, bacterial communities profiling is a critical next step toward understanding mosquito microbiome modulation and its implications for vector control.

Research on bacterial symbionts already demonstrates practical applications. *Wolbachia* transinfections have achieved stable field establishment and potent virus-blocking effects in *Aedes* (Moreira et al., 2009; Walker et al., 2011). Paratransgenic strategies using *Asaia*, *Serratia*, or *Chromobacterium* have also shown promise against *Plasmodium* and arboviruses (Wang et al., 2011; Chouaia et al., 2012; Ramirez et al., 2014; Favia et al., 2007). Characterizing the bacterial communities of sylvatic mosquito assemblages is thus not merely descriptive but a foundation for identifying locally abundant symbionts that can be tested for functional applications.

Developing innovative control strategies requires deeper knowledge of natural mosquito populations and their microbiota. In Colombia, studies of non-urban Culicinae have begun to document bacterial and viral diversity, with insect-specific viruses taxa reported in tribes such as Culicini and Sabethini (Gómez-Palacio et al., 2025). Although intensified malaria control efforts have reduced incidence in parts of Latin America, transmission persists due to ecological reservoirs, heterogeneous regional trends, and localized epidemic increases (Rovira-Vallbona et al., 2017; MacDonald and Mordecai, 2019; Grillet et al., 2021). Similar complexities shape arbovirus circulation, where urban vectors intersect with sylvatic reservoirs under conditions of climate change and land-use transformation. Against this backdrop, microbiome-informed interventions—including symbiont replacement, paratransgenesis, and bacterial communities’ modulation—offer innovative complements to conventional vector control.

Here, we investigate the bacterial communities of rural Culicinae mosquitoes from two localities in Antioquia, Colombia, to establish a baseline for microbiome-modulation strategies. Using metagenomic sequencing, we applied two complementary approaches: (i) extraction and profiling of 16S ribosomal sequences from total RNA-seq data to assess bacterial community diversity, and (ii) assembly of metagenomic contigs with taxonomic annotation against the NCBI NR bacterial database to expand resolution and functional insight. We hypothesized that sylvatic Culicinae harbor distinct bacterial assemblages shaped by host species and locality, including symbionts with potential relevance for vector competence and pathogen suppression. By integrating bacterial communities’ analysis with prior virome profiling of the same mosquito communities, this study provides a comprehensive microbial perspective on non-urban Colombian Culicinae. Our findings highlight the bacterial communities as a reservoir of candidate symbionts and a foundation for innovative, sustainable strategies to mitigate the burden of mosquito-borne diseases.

## Materials and methods

### Mosquito sampling and processing

Mosquito collections used in this study were mostly the same to those previously described for virome characterization of Colombian Culicinae (Gómez-Palacio et al., 2025). In brief, a total of 182 adult mosquitoes were collected from two rural localities in Antioquia, Colombia: the Río Claro River basin in Puerto Triunfo (Central Cordillera, 300 m a.s.l.) and the dry forest of Santa Fe de Antioquia (510 m a.s.l.) during November–December 2020. Sampling combined active methods (mouth aspirators and entomological nets) with passive traps (CDC white LED light traps and Shannon light traps).

Captured mosquitoes were individually transferred into 1.5 mL Eppendorf tubes containing 1 mL RNAlater® (Sigma-Aldrich, St. Louis, USA), maintained at ~4 °C in a portable cooler, and transported to the laboratory to preserve nucleic acids. To minimize bias from exogenous microbial material, fed females were excluded. Feeding status was determined under a stereoscope prior to species-level identification. Morphological identification followed standard taxonomic keys on a frozen surface to ensure both accuracy and RNA preservation. Mosquitoes were then sorted into species/morphotype groups and organized into 24 pools for downstream metagenomic analyses (Table 1). Collection of specimens was performed with landowner authorization and in accordance with Colombian environmental regulations (Decree No. 1376 of 2013, Ministry of Environment and Sustainable Development).

**Table 1:** Tribe, sample code, number of mosquitoes, origin, and species/morphotype of mosquitoes analyzed in this study.

Tribe	Sample name	No. of mosquitoes	Locality	Coordenates	Species/morphotype
Aedini	Ad_m08	4	San Francisco	5° 53′25” N 74° 51′30” W	*Ochlerotatus* sp. 1
	Ad_m09	4			*Ochlerotatus* sp. 2
	Ad_m18	3	Santa Fe de Antioquia	6° 31′49” N 75° 49′40” W	*Aedes albopictus*
	Ad_m19	11		6° 31′55” N 75° 49′57” W	
	Ad_m20	9			
	Ad_m21	6		6° 32′16” N 75° 49′51” W	*Aedes* sp.
Culicini	Cx_m01	10	San Francisco	5° 53′50” N 74° 51′34” W	*Culex* sp. 1
	Cx_m02	12			*Culex* sp. 1
	Cx_m04	14			*Culex* sp. 1
	Cx_m12	10		5° 53′25” N 74° 51′30” W	*Culex* sp. 2
	Cx_m13	10			*Culex* sp. 3
	Cx_m14	5		5° 52′56” N 74° 51′28” W	*Culex* sp. 4
	Cx_m15	4			*Culex* sp. 5
	Cx_m17	18	Santa Fe de Antioquia	6° 33′51” N 75° 49′48” W	*Culex* sp. 1
	Cx_m24	5	San Francisco	5° 53′38” N 74° 51′46” W	*Culex* sp.
	Cx_m25	5			*Culex* sp.
	Cx_m26	6			*Culex* sp.
Orthopodmyiini	Or_m22	5	Santa Fe de Antioquia	6° 32′16” N 75° 49′51” W	*Orthopodomyia* sp.1
	Or_m23	7			*Orthopodomyia* sp. 2
Sabethini	Sa_m05	10	San Francisco	5° 53′25” N 74° 51′30” W	*Wyeomyia* sp. 1
	Sa_m06	3			*Sabethes* sp.
	Sa_m07	4			*Trichoprosopon* sp.
	Sa_m10	8			*Wyeomyia* sp. 2
	Sa_m11	9			*Wyeomyia* sp. 3

### RNA extraction, library preparation, and sequencing

Total RNA was purified from the aqueous phase of TRIzol extracted samples using the High Pure Viral Nucleic Acid Kit (Roche Diagnostics, Mannheim, Germany) following the manufacturer’s instructions. To eliminate contaminating DNA, extracted nucleic acids were treated with DNase I (Thermo Fisher Scientific) according to the manufacturer’s protocol. RNA quality was assessed using an Agilent 2,100 Bioanalyzer (Agilent Technologies), and only samples with an RNA integrity number (RIN) > 7 were processed further. All RNA samples had concentrations exceeding 100 ng/μL, ensuring sufficient input material for library preparation and sequencing. Libraries were prepared using the TruSeq Total RNA Library Preparation Kit (Illumina) with random hexamer priming, and sequenced on a NovaSeq 6,000 S4 system (Illumina) with a 150-bp paired-end protocol at the Helmholtz Centre for Infection Research (HZI, Braunschweig, Germany).

### Raw data processing, bacteria identification and taxonomic annotation

**Figure 1:**
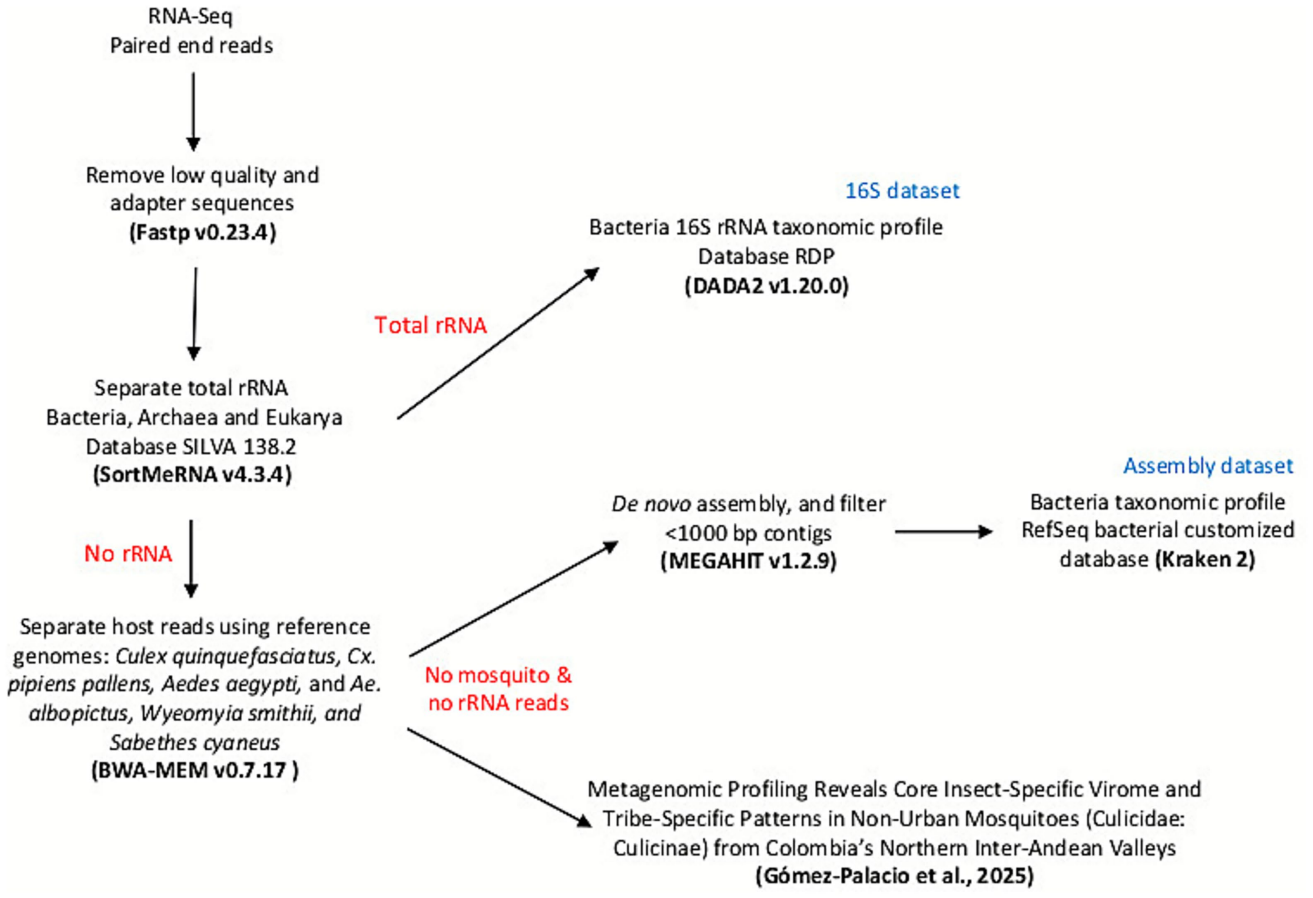
Schematic representation of the RNA-seq data processing pipeline. Clean reads were then divided into ribosomal RNA (rRNA) and non-rRNA fractions using SortMeRNA against SILVA reference sequences. The rRNA fraction (16S dataset) was processed with DADA2 for ribosomal taxonomic profiling of bacterial communities. Non-rRNA reads were mapped to available mosquito reference genomes with BWA-MEM to remove host sequences, and the remaining reads were assembled *de novo* with MEGAHIT. Contigs shorter than 1,000 bp were discarded, and the resulting assembly dataset was taxonomically annotated with Kraken2 against a custom bacterial database built from NCBI RefSeq genomes.

The RNA-seq processing pipeline is summarized in Figure 1. Raw paired-end reads were quality-filtered and adapter-trimmed with fastp v0.23.4 (Chen et al., 2018), retaining sequences with Phred scores > 20. Ribosomal RNA (rRNA) sequences were then identified against non-redundant bacterial, archaeal, and eukaryotic references from the SILVA 138.2 database (Quast et al., 2012) using SortMeRNA v4.3.4 (Kopylova et al., 2012) with an e-value cutoff of 1 × 10^−5^. The rRNA fraction (hereafter 16S dataset) was retained for direct taxonomic profiling of bacterial communities using RDP database (Callahan et al., 2016). These reads were processed in R with the DADA2 v1.20.0 package (Callahan et al., 2016) to denoise sequences, infer amplicon sequence variants (ASVs), and generate abundance tables suitable for downstream diversity analyses.

Reads not assigned as rRNA were mapped against available mosquito reference genomes (*Culex quinquefasciatus* GCF_015732765.1, *Cx. pipiens pallens* GCF_016801865.2, *Aedes aegypti* GCF_002204515.2, *Ae. albopictus* GCF_006496715.1, *Wyeomyia smithii* GCF_029784165.1, and *Sabethes cyaneus* GCF_943734655.1) using BWA-MEM v0.7.17 (Li and Durbin, 2010; Li, 2013) to remove residual host sequences. The resulting non-host, non-rRNA reads were *de novo* assembled with MEGAHIT v1.2.9 (Li et al., 2015), and contigs shorter than 1,000 bp were discarded. This produced a second dataset (hereafter assembly dataset), composed of longer bacterial contigs that complement ribosomal profiles with broader genomic signals. Taxonomic classification of contigs was performed with Kraken2 (Wood et al., 2019), using the NCBI RefSeq bacterial database (Release 200, August 26, 2020) as the reference.

This metatranscriptomic 16S approach leveraged the same total RNA-seq libraries used for virome profiling, enabling simultaneous detection of bacterial and viral components. Because these libraries were prepared with random hexamer priming and rRNA depletion, the recovered 16S fragments were short (≈150–300 bp), limiting taxonomic resolution compared with targeted amplicon or long-read sequencing.

Together, these two datasets—(i) 16S rRNA-derived ASVs and (ii) assembled bacterial contigs—provided complementary perspectives for bacterial community’s characterization, enabling both fine-scale taxonomic profiling and broader genomic context.

### Bacteria abundance and diversity based on 16S dataset

For 16S dataset; no ambiguous bases were permitted (maxN = 0), maximum expected errors were limited to two per read (maxEE = c (2,2)), and bases with quality scores below Q2 were trimmed (truncQ = 2); phiX contaminants were eliminated (rm.phix = TRUE). After sequence inference, paired reads were merged, and chimeras were removed using the “consensus” method in removeBimeraDenovo. To reduce noise, we discarded any ASV with fewer than 100 total reads across all samples.

The resulting ASV table was analyzed in phyloseq v1.36.0 (McMurdie and Holmes, 2013). Taxonomy was assigned against the RDP training set version 19 for DADA2 (Callahan et al., 2016) using the assignTaxonomy and addSpecies functions. To control for sequencing depth, samples were rarefied to the minimum read count retained after filtering, and rarefaction curves were generated with rarecurve (step = 10) in vegan v2.6.4 (Oksanen et al., 2022) to confirm sampling sufficiency.

Normalized relative abundances were obtained with the transform_sample_counts function in phyloseq (total sum scaling, TSS) for downstream analyses. Alpha (*α*) diversity was estimated with Chao1 richness and Shannon–Weaver diversity indices, and pairwise differences were tested using Kruskal–Wallis tests. Beta (*β*) diversity was calculated from Bray–Curtis dissimilarities to evaluate compositional variation among mosquito species/morphotypes, and significance was assessed by PERMANOVA (permutation test with pseudo-F ratios) using the adonis function in vegan v2.7.1 (Oksanen et al., 2022).

### Bacterial community’s abundance based on assembly dataset

Contigs classified as bacterial taxa by Kraken2 were retained and used as mapping references. Paired-end RNA reads from each sample were mapped back to bacterial contigs using Bowtie2 v2.5.1 (Langmead et al., 2019), and sorted BAMs were produced with Samtools v1.9 (Li et al., 2009; Danecek et al., 2021). Per-contig read counts were extracted with *samtools idxstats* and converted to RPKM (reads × 10^9^ / contig_length / total_mapped_reads) to normalize for contig length and library size. Counts were aggregated to taxonomic identifiers reported by Kraken2 (Wood et al., 2019). For visualization, taxon abundances were normalized to relative abundance per sample, low-abundance taxa were collapsed into “Other” (top-N approach), and stacked barplots were generated in R using ggplot2 and ggh4.

### Differential abundance and clustering analyses

To evaluate differences in microbiome composition across mosquito morphotypes, differential abundance testing was performed with ANCOM-BC (Lin and Peddada, 2020) in R. Analyses were conducted at the genus level following prevalence and abundance filtering and sample-level quality control. Significant taxa were extracted and visualized in clustered heatmaps with sample annotations using the ComplexHeatmap package (Gu et al., 2016).

Bacterial communities clustering and group differentiation were further examined using Discriminant Analysis of Principal Components (DAPC), implemented with the adegenet package in R (Jombart, 2008). The resulting Linear Discriminant (LD) scores were plotted with 95% confidence ellipses to illustrate group differentiation. Ordination plots generated through the LDAKPC framework provided a visual representation of bacterial communities clustering across mosquito species and morphotype pools.

## Results

### Processing data output and dataset assembly

After sequencing, a total of 403.7 million clean reads were obtained across 24 mosquito pools, with an average of 1.68 × 10^7^ reads per pool (SD ± 1.33 × 10^7^) (Supplementary Table S1). From these, the 16S dataset comprised 673,044 paired reads (mean 28,044 ± 30,985 per pool), while the complementary assembly dataset yielded 60,962 contigs >1 kb (mean 2,540 ± 2,692 per pool). Assembly output varied among tribes: Aedini pools generated 1,599–3,703 contigs, Culicini displayed the widest heterogeneity, ranging from as few as 23 contigs (Cx_m04) to 12,823 (Cx_m17), Orthopodomyini produced moderate assemblies (691–1,781 contigs), and Sabethini ranged from 1,386 in *Wyeomyia* sp. (Sa_m05) to 4,964 in *Sabethes* sp. (Sa_m06). Non-parametric testing showed no significant differences in 16S rRNA read counts among tribes (Kruskal–Wallis χ^2^₃ = 0.97, *p* = 0.81), a result consistent with parametric analysis (ANOVA: F₃,20 = 0.50, *p* = 0.68). Similarly, contig counts from the assembly dataset did not differ significantly across tribes (Kruskal–Wallis χ^2^₃ = 4.01, *p* = 0.26; ANOVA: F₃,20 = 0.22, *p* = 0.88). Together, these results indicate that sequencing depth and assembly yields were broadly comparable across Aedini, Culicini, Orthopodmyiini, and Sabethini pools, with no statistically supported tribe-level differences.

Rarefaction analysis confirmed that sequencing depth was sufficient to capture the majority of bacterial diversity across mosquito pools (Supplementary Figure S1). To standardize diversity comparisons, all libraries were rarefied to 6,760 paired-end reads, corresponding to the lowest depth retained after quality filtering (Supplementary Table S1). These results confirm both the adequacy of sequencing effort and the broad variability in retained bacterial signals across mosquito tribes, providing a robust foundation for downstream diversity and taxonomic analyses.

### Taxonomic composition of the mosquito bacterial communities based on 16S dataset

Across all mosquito tribes, a considerable fraction of 16S rRNA reads could not be resolved beyond higher taxonomic ranks. The proportion of assigned reads ranged from 8.1% in Aedini to 14.1% in Culicini, while Orthopodmyiini and Sabethini showed intermediate values (7.9 and 11.4%, respectively). Unclassified reads were distributed across all tribes, with a large proportion remaining unresolved at higher taxonomic levels (81.3% in Aedini, 76.7% in Orthopodmyiini, 75.7% in Sabethini, and 71.1% in Culicini). A smaller fraction of reads could not be confidently assigned at the genus level, representing 15.3% in Orthopodmyiini, 15.7% in Culicini, 12.9% in Sabethini, and 10.6% in Aedini. The high proportion of unclassified reads (>70%) most likely reflects the short and fragmented nature of bacterial 16S sequences recovered from host-derived RNA-seq libraries, rather than limitations of the RDP database or DADA2 parameters. These patterns underscore both the challenges of classifying mosquito-associated bacterial sequences in rural Neotropical populations and the importance of reporting complementary contig-based annotations to improve taxonomic resolution.

**Figure 2:**
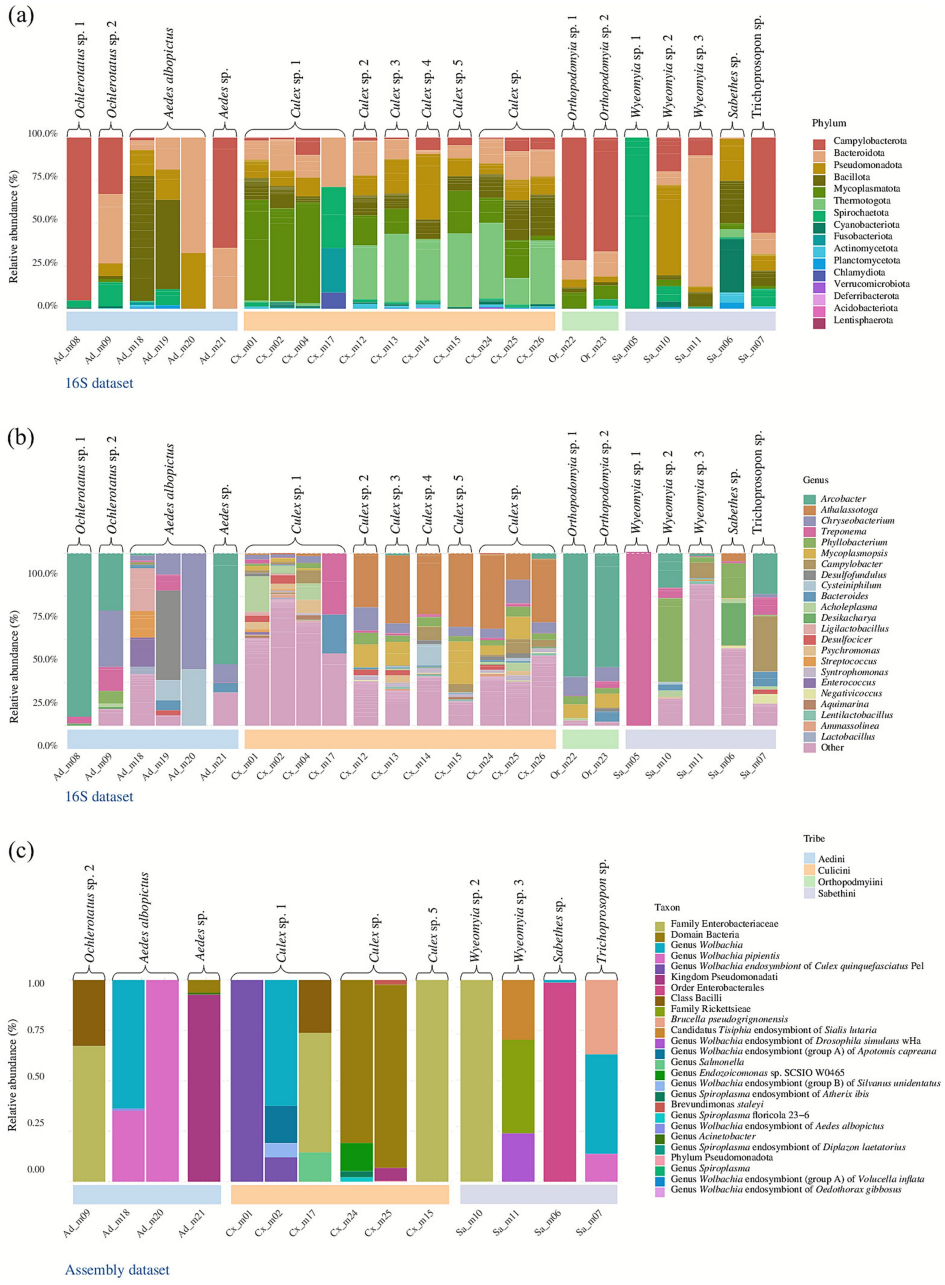
Taxonomic composition of the mosquito bacterial communities. Relative abundance of bacterial taxa across mosquito tribes based on **(a)** 16S dataset at the phylum level, **(b)** 16S dataset at the genus level, and **(c)** assembly dataset annotated to the maximum taxonomic resolution. While 16S data highlighted broad phylum- and genus-level patterns, assembly-based profiling revealed dominant endosymbionts (*Wolbachia*) alongside environmental and commensal taxa.

The 16S dataset revealed a diverse bacterial community spanning 18 phyla, 104 families, and over 171 genera (Supplementary Tables S2, S3). At the phylum level, Bacillota dominated, accounting for nearly 50.4% of total reads, followed by Bacteroidota (17.6%) and Pseudomonadota (12%). Other phyla such as Mycoplasmatota, Campylobacterota, Thermotogota, Cyanobacteriodita, and Spirochaetota contributed smaller but consistent fractions across samples, while Planctomycetota, Verrucomicrobiota, and Fusobacteriota were detected at lower abundance (Figure 2a). At the family level, the most represented groups included Flavobacteriaceae, Peptostreptococcaceae, Mycoplasmataceae, Candidatus_*Carsonella*, and Mesoaciditogaceae, which together comprised a substantial proportion of the bacterial communities. Additional families of ecological and medical interest were also detected, including Enterobacteriaceae, Francisellaceae, Lactobacillaceae, and Mycoplasmataceae (Supplementary Tables S2, S3).

At the genus level, several endosymbiotic or vector-relevant taxa were identified. These included *Borrelia*, *Mycoplasmopsis*, *Athalassotoga*, *Arcobacter*, and *Spiroplasma*, all known as intracellular symbionts of insects. Other genera of interest included *Acinetobacter* (Moraxellaceae), *Enterococcus* and *Lactobacillus* (Firmicutes), *Streptococcus*, *Klebsiella*, *Helicobacter*, and *Escherichia*–*Shigella*, suggesting both commensal and potentially pathogenic associations. (Figure 2b).

### Bacterial community’s characterization based on the assembly dataset

Taxonomic assignment of assembled contigs provided higher-resolution annotations than the 16S dataset, frequently reaching the genus or symbiont-specific level (Figure 2c). Overall, the assembly dataset revealed the predominance of endosymbiotic and commensal lineages, with a marked dominance of *Wolbachia* across multiple mosquito tribes. In addition to *Wolbachia*, other taxa of potential medical or ecological relevance were detected. These included *Rickettsiaceae* (e.g., *Brucella pseudogrignonensis*), *Spiroplasma* endosymbionts from multiple insect hosts (*Atherix ibis, Diplazon laetatorius*), and *Endozoicomonas* (Figure 2c). Environmental genera such as *Acinetobacter*, *Brevundimonas*, and *Salmonella* were also detected, though at lower relative abundances. Tribe-specific differences were evident: while Aedini pools were dominated by *Wolbachia*, Culicini pools showed more heterogeneous compositions including *Enterobacteriaceae* and *Pseudomonadota*, and Sabethini pools contained mixed assemblages with *Wolbachia*, *Spiroplasma*, and *Endozoicomonas*.

### *α*-diversity of bacterial communities across mosquito species/morphotypes

**Figure 3:**
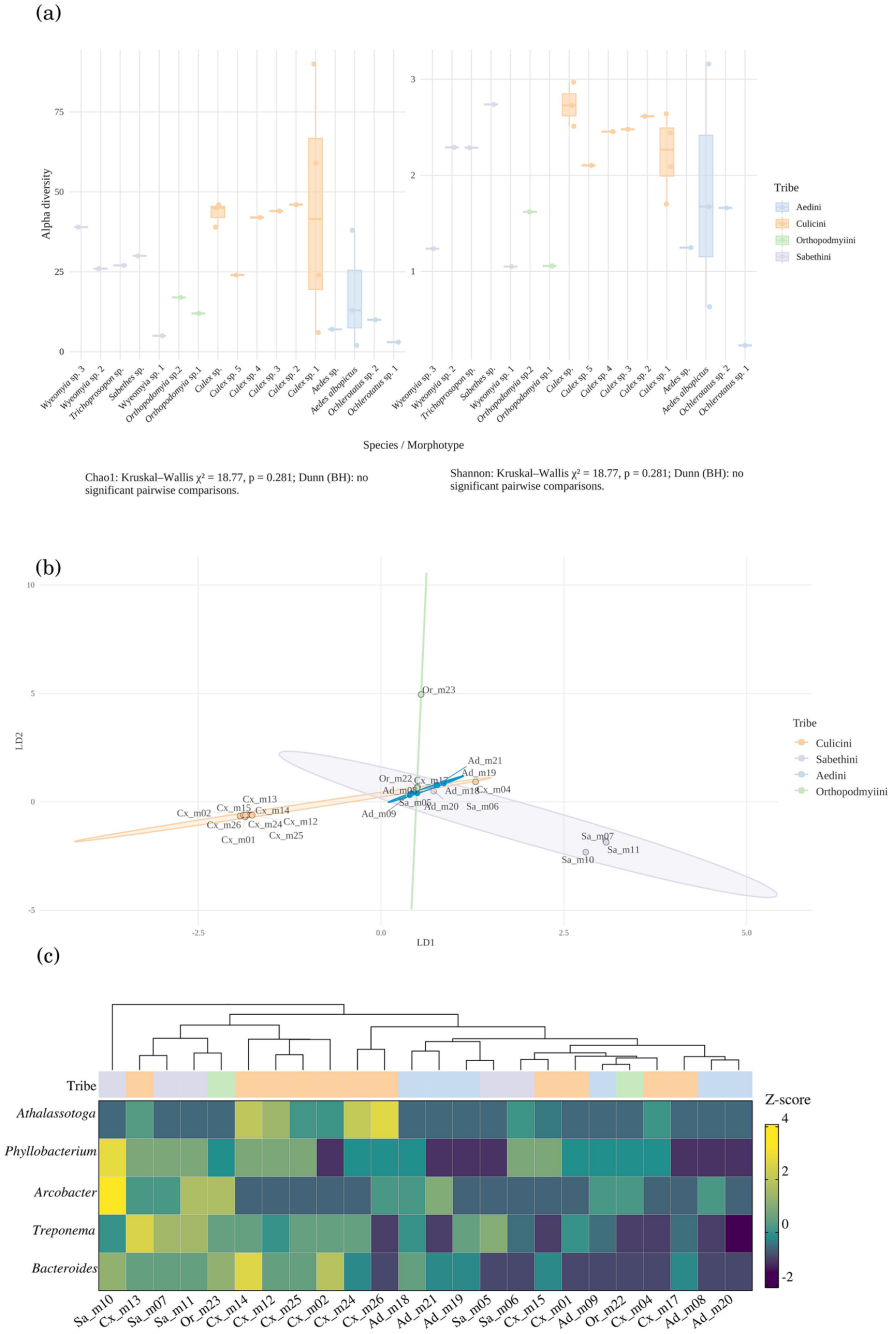
Diversity patterns of bacterial communities in Culicinae morphotypes from Antioquia, Colombia**. (a)** *α*-Diversity (Chao1 richness, Shannon diversity) grouped by species/morphotype; no significant differences detected (*p* > 0.05). **(b)** *β*-Diversity (DAPC) showing partial clustering by species/morphotype; *Culex* overlapped with *Aedes*, while *Ochlerotatus*, *Wyeomyia*, and *Sabethes* were more distinct. **(c)** Heatmap of differentially abundant genera, with Aedini enriched in lactic acid bacteria and Culicini showing broader endosymbiont- and environment-associated taxa.

The Chao1 (richness) and Shannon (evenness) indices revealed variation in bacterial communities’ diversity across mosquito species/morphotypes (Figure 3a). *Culex* specimens consistently exhibited the highest median values for both α-diversity indices. Intermediate diversity values were observed in *Wyeomyia*, *Sabethes*, and some *Ochlerotatus,* and *Orthopodomyia* pools, while *Trichoprosopon* and some *Aedes* pools displayed the lowest diversity. However, global non-parametric tests indicated no statistically significant differences among morphotypes (Kruskal–Wallis, Chao1: χ^2^ = 18.77, *p* = 0.281; Shannon: χ^2^ = 18.77, p = 0.281). Pairwise post-hoc comparisons (Dunn test with Benjamini–Hochberg correction) also failed to identify significant differences between specific morphotype pairs (adjusted *p* > 0.05).

### *β*-diversity and species/morphotype-level differentiation

β-diversity was first estimated using Bray–Curtis dissimilarities, which revealed moderate compositional differences among mosquito species and morphotypes. These distances were further explored through DAPC, which showed partial grouping of bacterial community composition according to species/morphotype (Figure 3b). The first two discriminant axes explained nearly all the discriminant variance (LD1 = 62.4%, LD2 = 35.7%). Along LD1, *Orthopodomyia* sp. 2 was clearly separated from all other groups, displaying the highest positive values on this axis. In contrast, *Orthopodomyia* sp. 1 (Or_m22) and *Ochlerotatus* clustered near the origin, alongside *Aedes albopictus* and other *Aedes*. Most *Culex* samples formed a central cluster, indicating compositional similarity and partial overlap with Aedini samples. The Sabethini tribe showed greater dispersion, with *Wyeomyia* and *Trichoprosopon* distributed along the negative values of LD1 and LD2, while *Sabethes* was positioned close to the central cluster. Overall, the combined Bray–Curtis and DAPC analyses highlight clear compositional separation for *Orthopodomyia* and Sabethini members, while *Culex* and *Aedes* exhibit substantial overlap, reflecting high within-group similarity.

### Differential abundance of bacterial communities in sylvatic mosquitoes from Colombia

Genus-level differential-abundance analyses revealed strong heterogeneity in bacterial communities’ composition across mosquito morphotypes (Figure 3c). The most pronounced contrast was between *Aedes albopictus* and *Culex* spp., with 5 genera showing significant shifts. Orthopodmyiini and Sabethini generally display higher z-scores for *Athalassotoga*, *Phyllobacterium*, and *Arcobacter* responsible of the latter being an emerging genus of zoonotic bacteria associated with gastroenteritis in humans and abortions in animals. Culicini samples tend to cluster together and exhibit intermediate or lower abundances for these taxa. Aedini samples show more variable profiles, with *Bacteroides* and *Treponema* presenting higher z-scores in select individuals. The hierarchical clustering above the heatmap further highlights tribe-level groupings, suggesting that bacterial community composition is more similar within tribes than between them. Overall, the heatmap underscores tribe-specific patterns in bacterial genus abundance, while also revealing substantial heterogeneity among individual samples within the Aedini and Sabethini tribes.

## Discussion

Our findings support the initial hypothesis that sylvatic *Culicinae* mosquitoes harbor distinct bacterial assemblages shaped by host species and locality. Both the 16S and assembly-based datasets revealed taxonomically diverse microbiomes dominated by *Wolbachia* and other symbionts, along with environmental and potentially pathogenic taxa that varied across tribes and sampling sites. These results indicate that host lineage and ecological context jointly influence bacterial community structure, with several detected symbionts—such as *Wolbachia* and *Spiroplasma*—known to play roles in pathogen interference and microbiome modulation.

This study provides the first detailed characterization of bacterial communities associated with rural *Culicinae* mosquitoes from two localities in Antioquia, Colombia. Overall, the microbiome was dominated by *Pseudomonadota* but also included symbiotic, environmental, and potentially pathogenic taxa spanning more than a dozen bacterial phyla, underscoring its taxonomic breadth and ecological complexity (Coon et al., 2014b, 2016; Guégan et al., 2018). The assembly-based analysis revealed high proportions of *Wolbachia* reads in several *Aedes* (e.g., Ad_m18, Ad_m20) and *Culex* morphotypes (e.g., Cx_m24, Cx_m25), often corresponding to known host–symbiont associations such as *Wolbachia pipiens* (endosymbiont of *Culex quinquefasciatus*) and *Wolbachia* of *Aedes albopictus*. In contrast, the 16S dataset derived from RNA-seq provided broader but lower-resolution community profiles, reflecting the inherent limitations of host-derived total RNA libraries. Because bacterial ribosomal sequences occur at low abundance and are frequently fragmented or partially removed during rRNA depletion, only partial 16S regions are recovered, reducing the accuracy of taxonomic assignment compared with targeted amplicon or metagenomic sequencing. Together, these complementary approaches highlight the coexistence of dominant intracellular symbionts and low-abundance environmental taxa, supporting a model where host lineage and ecological context jointly structure the bacterial communities (Dong et al., 2009b; Coon et al., 2014b; Hegde et al., 2015; Guégan et al., 2018).

Patterns of *α*-diversity (Chao1 and Shannon indices) did not show significant differences among species/morphotypes, but *β*-diversity analyses (PERMANOVA *p* = 0.001) revealed clear clustering by host group. Notably, *Aedes albopictus Streptococcus* was detectable and other lactic acid bacteria such as *Ligilactobacillus*, *Lactobacillus* and *Enterococcus* were present at low abundance or absent; instead, these individuals were dominated by non-lactic taxa (e.g., Chryseobacterium, Treponema), suggesting that LAB-mediated gut homeostasis and antiviral interference may be less pronounced in these populations. In contrast, *Culex* morphotypes carried a broader array of endosymbionts and environmental taxa, including members of Rickettsiales and insect-associated symbionts such as *Spiroplasma*, as well as diverse environmental lineages that frequently appear in metagenomic surveys of *Culex* (Guégan et al., 2018; Wang et al., 2024; Calle-Tobón et al., 2022; Pollio et al., 2022). This dichotomy is consistent with previous reports of host-specific microbial associations, and suggests that Aedini and Culicini may differ not only in microbiome composition but also in the functional contributions of their bacterial partners to vector competence.

Beyond taxonomic contrasts, our data indicate the presence of a relatively homogeneous “core” bacterial communities composed of abundant, recurrent taxa shared across multiple species/morphotypes, complemented by a long tail of rare taxa. While the core likely provides stable ecological functions within the mosquito microbiome, it is the rare taxa that contribute disproportionately to differentiation among species and sites (Moreira et al., 2009; Guégan et al., 2018). These low-frequency bacteria may act as sensitive indicators of local microhabitats, feeding history, or fine-scale host–environment interactions, and thus carry the strongest signal of coevolution between mosquitoes and their associated microbiota (Wang et al., 2011; Coon et al., 2014b; Guégan et al., 2018).

Non-urban mosquitoes can act as carriers of bacteria relevant for human, veterinary, and even plant health, either as mechanical/biological vectors or indirectly by modulating arbovirus transmission. The presence of such bacteria in sylvatic populations highlights their potential role in rural transmission cycles, where mosquitoes may bridge microbial interactions across wildlife, domestic animals, and humans (Golding et al., 2015; Ramasamy and Surendran, 2016; Cano-Pérez et al., 2023). This finding emphasizes the need to consider microbiota not only as modulators of arboviral competence but also as potential reservoirs of opportunistic pathogens in rural landscapes.

Analysis of the 16S dataset revealed several bacterial taxa of recognized clinical, veterinary, and ecological importance in rural Culicinae mosquitoes. Enteric pathogens such as *Salmonella*, a zoonotic agent responsible for global salmonellosis outbreaks (Galán-Relaño et al., 2023), and *Clostridium perfringens*, a frequent cause of foodborne intoxication (Grenda et al., 2023), highlight the potential for mosquitoes to harbor bacteria relevant to food safety. Vector-borne pathogens were also detected, *Borrelia* spp. (Steere et al., 2016), underscoring the zoonotic dimension of the bacterial communities. Opportunistic and nosocomial taxa such as *Bacteroides fragilis*, *Enterococcus*, *Streptococcus*, and *Veillonella parvula* (Zhou et al., 2021) were present, alongside commensals with beneficial roles, including *Lactobacillus* and *Bifidobacterium*. Additional taxa such as *Cutibacterium acnes* and *Haemophilus parainfluenzae* were identified as potential opportunists. Finally, the widespread symbiont *Wolbachia* was detected, reinforcing its relevance as a naturally occurring biocontrol agent in mosquitoes.

Several bacterial taxa identified in this study overlap with those already recognized as candidates for microbiome-based control strategies. *Wolbachia*, detected in both *Aedes* and *Culex*, has been widely used in biocontrol through transinfection and natural strain amplification, with well-documented virus-blocking effects in *Ae. aegypti,* and additional roles in reproductive manipulation, including male killing (MK) (Moreira et al., 2009; Walker et al., 2011; Caragata et al., 2016; Pan et al., 2018b; Arai et al., 2023; Zhang et al., 2024). Likewise, *Spiroplasma*, detected among *Culex*-associated taxa, is an insect endosymbiont known for inducing MK and conferring antiviral protection in dipterans (Arai et al., 2023). Its occurrence in our samples represents an additional candidate for microbiome-based interventions pending targeted functional validation (Karatepe et al., 2018; Shimooka et al., 2021). The differential enrichment of lactic acid bacteria in Aedini versus broader environmental taxa in Culicini suggests distinct ecological reservoirs of candidate symbionts. However, translating these associations into control strategies requires careful functional validation, ideally through targeted qPCR assays, transinfection experiments, or controlled infection trials. These results demonstrate how bacterial communities profiling in sylvatic mosquitoes can inform the identification of biocontrol candidates.

Our previous virome analysis in the same mosquito pools revealed a high proportion of bacteriophages, which likely interact with the bacterial fraction of the microbiome (Vasilakis and Tesh, 2015; Shi et al., 2020; Moonen et al., 2023; Gómez-Palacio et al., 2025). Phages can regulate bacterial abundance, alter competitive dynamics, and influence the stability of mosquito-associated communities (Shi et al., 2020). Conversely, bacterial community composition creates niches for specific phages, shaping the broader virome (Shi et al., 2020; Moonen et al., 2023). While our study did not explicitly test phage–bacteria interactions, the co-occurrence of bacterial communities and virome data suggests a multilayered network where phages may mediate microbiome modulation. Integrating ASV–vOTU co-occurrence analyses could provide a promising path to uncover non-causal but ecologically relevant associations, strengthening the hypothesis that bacteriophages are critical links between bacterial symbionts and mosquito vector competence.

Our findings partially align with prior reports on sylvatic and urban mosquitoes in Colombia and Latin America (Calle-Tobón et al., 2022; Cano-Pérez et al., 2023; Ramírez et al., 2024). For example, the higher richness observed in *Culex* is consistent with earlier studies documenting more complex bacterial assemblages in this genus, especially in rural or aquatic habitats (Wang et al., 2011; Guégan et al., 2018). The detection of *Wolbachia* in both *Aedes* and *Culex* contrasts with some local surveys that reported variable prevalence, possibly reflecting ecological differences between urban and rural habitats (Calle-Tobón et al., 2022; Cano-Pérez et al., 2023). Likewise, the enrichment of lactic acid bacteria in *Ae. albopictus* resonates with studies showing that these taxa can interfere with arboviral replication, supporting their candidacy for symbiont-based interventions (Chouaia et al., 2012; Ramirez et al., 2014). By situating our results within this broader context, we reinforce the novelty of characterizing bacterial communities in non-urban Colombian mosquitoes and highlight both convergences and divergences with previously described patterns. Our results suggest that sylvatic mosquito microbiomes in these rural sites differ substantially from previously reported “core” assemblages. This divergence likely reflects strong environmental and life-stage sourcing of the adult gut community and may have functional consequences for vector competence. We therefore recommend complementing 16S surveys with longitudinal sampling, shotgun metagenomics/metatranscriptomics and targeted functional assays (e.g., transinfection and virus-challenge experiments) to validate whether compositional differences translate into altered transmission risk.

Several caveats should be acknowledged when interpreting our results. First, replication was uneven across morphotypes, with some groups represented by only one or two pools, which reduces statistical power. Pooling individuals by species and site, while necessary for sequencing feasibility, may also obscure individual-level variation. Second, rarefying to the minimum sequencing depth avoided biases from uneven library sizes but may have excluded rare taxa that carry ecological signal. Third, our reliance on total RNA and rRNA extraction (SortMeRNA-based metataxonomy) captures metabolically active fractions but may be sensitive to environmental RNA contamination or biased toward highly expressed taxa. This approach also yields short, fragmented 16S regions, which reduces taxonomic resolution and likely explains the high proportion of unclassified reads. Finally, differential-abundance analyses are sensitive to multiple-testing correction and compositional effects, underscoring the need for validation via targeted approaches such as qPCR or shotgun metagenomics. In addition, our use of RNA-seq-derived 16S reads instead of targeted amplicon sequencing imposes intrinsic constraints on taxonomic resolution, as only partial 16S fragments are recovered from host total-RNA libraries. This limitation likely contributes to the high proportion of unclassified reads and should be considered when interpreting fine-scale bacterial assignments. Despite these limitations, the congruence between the 16S and assembly datasets strengthens confidence in our major conclusions.

By establishing a baseline characterization of bacterial communities in rural Culicinae mosquitoes from Antioquia, our study contributes directly to the development of microbiome-based strategies for disease control. Identifying symbionts such as *Wolbachia*, *Spiroplasma,* lactic acid bacteria, and other candidate taxa provides a pipeline for evaluating their functional role in vector competence and their potential utility in paratransgenic interventions. More broadly, integrating bacterial communities and virome perspectives offers a powerful framework to understand how microbial communities shape mosquito biology and pathogen transmission. These results underscore the importance of expanding entomological surveillance in non-urban habitats, where sylvatic mosquitoes intersect with human populations and contribute to the emergence of zoonotic diseases. Under this picture, our findings illustrate how microbiome profiling in natural mosquito populations can inform innovative, sustainable approaches to reduce the burden of mosquito-borne diseases in Latin America.

## Conclusion

This study provides the first comprehensive characterization of the bacterial communities in rural Culicinae mosquitoes from Antioquia, Colombia, revealing a diverse assemblage of symbiotic, environmental, and potentially pathogenic bacteria. We identified both a conserved core microbiome and a set of rare taxa that likely reflect fine-scale ecological interactions, host lineage, and microhabitat differences. The detection of *Wolbachia*, *Spiroplasma,* lactic acid bacteria, and other candidate symbionts underscores their potential relevance for microbiome-based control strategies, while the presence of opportunistic pathogens highlights the broader public health implications of sylvatic mosquito populations. By integrating bacterial communities and virome perspectives, our work establishes a baseline for understanding how microbial communities shape mosquito biology and vector competence in non-urban settings, and provides a foundation for developing sustainable, ecology-informed approaches to reduce the burden of mosquito-borne diseases in Latin America.

## Data Availability

The raw sequencing and identified ASV datasets used in the present study are available in the NCBI Sequence Read Archive under the BioProject accession PRJNA1199888.
