# Supplementary figures and images for: Detection of endosymbiotic, environmental, and potential bacterial pathogens in diverse mosquito taxa from Colombian tropical forests using RNAseq
Source: Front Microbiol. 2025 Dec 17;16:1727830. doi: 10.3389/fmicb.2025.1727830 (PMC12755158; doi:10.3389/fmicb.2025.1727830)

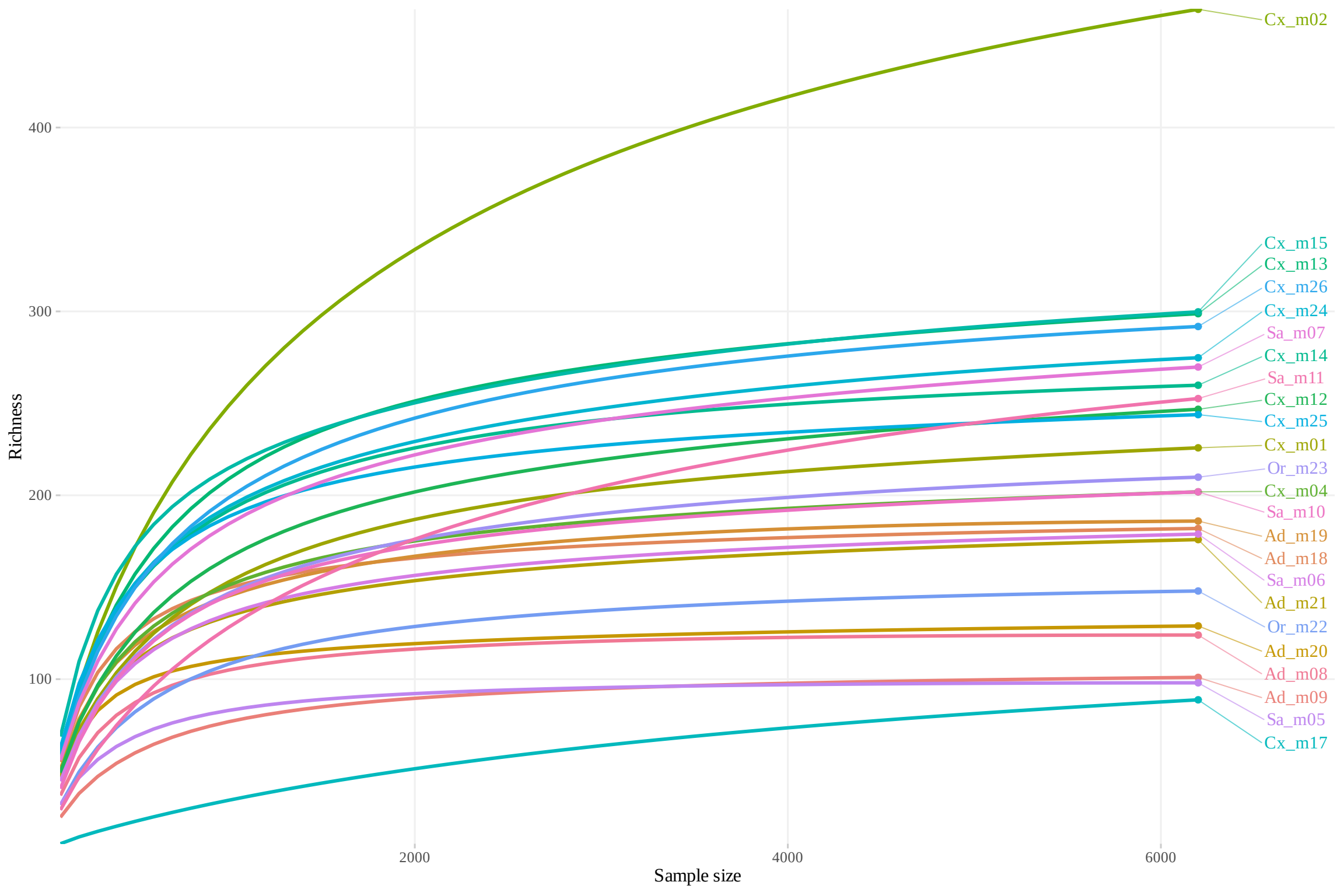

Supplement: Supplementary file 1 [file Data_Sheet_1.pdf]
